# *Candida* causes recurrent vulvovaginal candidiasis by forming morphologically disparate biofilms on the human vaginal epithelium

**DOI:** 10.1016/j.bioflm.2023.100162

**Published:** 2023-10-22

**Authors:** Yihong Pan, Yao Sun, Lanqian Chen, Yali Cheng, Panpan Jin, Weidan Zhang, Lingzhi Zheng, Junyan Liu, Tieli Zhou, Zhenbo Xu, Cheng Li, Xenia Kostoulias, Cathy J. Watson, David McGiffin, Anton Y. Peleg, Yue Qu

**Affiliations:** a Wenzhou Medical University-Monash BDI Alliance in Clinical and Experimental Biomedicine, The First Affiliated Hospital of Wenzhou Medical University, Zhejiang, 325000, China; b Department of Obstetrics and Gynaecology, Taizhou Hospital of Wenzhou Medical University, Zhejiang, 318050, China; c Department of Laboratory Medicine, The First Affiliated Hospital of Wenzhou Medical University, Wenzhou, Zhejiang, 325000, China; d Department of Pathology, Taizhou Hospital of Wenzhou Medical University, Zhejiang, 318050, China; e School of Food Science and Engineering, Guangdong Province Key Laboratory for Green Processing of Natural Products and Product Safety, Engineering Research Center of Starch and Vegetable Protein Processing, Ministry of Education, South China University of Technology, Guangzhou, 510640, China; f Department of Civil and Environmental Engineering, University of Maryland, College Park, MD, 20742, United States; g Department of Biology, Massachusetts Institute of Technology, Cambridge, MA, 02139, United States; h Department of Infectious Diseases, The Alfred Hospital and Monash University, Melbourne, 3004, Australia; i Department of Microbiology, Infection Program, Biomedical Discovery Institute, Monash University, Clayton, 3800, Australia; j School of Population and Global Health, University of Melbourne, Carlton, 3053, Australia; k Department of Cardiothoracic Surgery, The Alfred and Monash University, Melbourne, Victoria, 3004, Australia

**Keywords:** Recurrent vulvovaginal candidiasis (RVVC), Patients, Biopsy, *Candida* biofilms, Histopathology, Antifungal resistance, And persistence

## Abstract

**Background:**

Recurrent vulvovaginal candidiasis (RVVC) is a recalcitrant medical condition that affects many women of reproductive age. The importance of biofilm formation by *Candida* in RVVC has been recently questioned. This study aimed to elucidate the fundamental growth modes of *Candida* in the vagina of patients with RVVC or sporadic vulvovaginal candidiasis (VVC) and to assess their roles in the persistence of RVVC.

**Methods:**

Vaginal tissues were sampled from twelve patients clinically and microbiologically diagnosed as RVVC or VVC at a post-antifungal-treatment and asymptomatic period. High-resolution scanning electron microscopy, fluorescence in situ hybridization in combination with *Candida*-specific 18S rRNA probes and viable fungal burden were used to qualitatively and quantitatively evaluate *Candida* growth in the human vagina. The presence of *Candida* biofilm extracellular polymeric substances was examined using confocal laser scanning microscopy and biopsy sections pre-stained with Concanavalin A. Histopathological analysis was carried out on infected vaginal tissues stained with hematoxylin and eosin. Lastly, the susceptibility of epithelium-associated *Candida* biofilms to fluconazole at the peak serum concentration was evaluated.

**Results:**

*Candida* species grew on the vaginal epithelium of RVVC patients as morphologically disparate biofilms including monolayers, microcolonies, and macro-colonies, in addition to sporadic adherent cells. *Candida* biofilm growth on the vaginal epithelium was associated with mild lymphocytic infiltration of the vaginal mucosa. These epithelium-based *Candida* biofilms presented an important characteristic contributing to the persistence of RVVC that is the high tolerance to fluconazole.

**Conclusions:**

In summary, our study provides direct evidence to support the presence of *Candida* biofilms in RVVC and an important role of biofilm formation in disease persistence.

## 1 Background

Recurrent vulvovaginal candidiasis (RVVC, defined as three or more symptomatic episodes in 12 months) is a difficult-to-cure fungal infection that significantly affects quality of life of many women of reproductive age [1,2], with a global annual prevalence of 3871 per 100,000 women reported [3]. *Candida albicans* is the major aetiological agent, causing at least three quarters of all RVVC cases [4,5]. Most *C. albicans* clinical isolates from patients with sporadic vulvovaginal candidiasis (VVC, less than three episodes in 12 months) and RVVC retain susceptibilities to first-line antifungals [6,7]. Patients with VVC generally respond well to short-term topical antifungal therapy or single-dose oral treatment [8,9]. RVVC patients, although initially respond to antifungal treatments recommended by The Centers for Disease Control and Prevention (CDC), the causative *Candida* cells often remerge rapidly and cause a new symptomatic episode upon discontinuation of antifungals [2,6,10].

The frequent ineffectiveness of first-line antifungal drugs in curing RVVC, despite the reported susceptibilities of *Candida* to these agents, suggests that fungal survival strategies other than intrinsic resistance might be involved [11]. Biofilm formation has been long proposed as an important virulence trait of *Candida* species that contributes to the occurrence and sub-optimal antifungal treatment of RVVC [11,12]. Cumulative experimental evidence from animal studies using murine models supports the important role of *Candida* biofilms in the pathogenesis of VVC/RVVC [[11], [12], [13]]. Murine models of VVC, however, do not precisely represent the human vaginal environment and differ from human VVC/RVVC in several physiological aspects, including a lack of *C. albicans* in the vaginal microbiota, neutral vaginal pH, and dependence on exogenous estrogen to initiate fungal colonization [12,14]. Such differences have led researchers to question whether the importance of *Candida* biofilm formation in RVVC established in rodents could be transferred to humans [[14], [15], [16]]. Swidsinski et al. recently examined vaginal tissues from VVC/RVVC patients using Fluorescence In Situ Hybridization (FISH) in combination with 18S rRNA probes specific for *Candida* cells and disputed the presence of *Candida* biofilms in the human vagina [17]. FISH with16S rRNA probe has been successfully used by the same group to detect bacterial biofilms grown on the human vaginal epithelium [18]. The sensitivity and specificity of 18S rRNA probe against clinical *Candida* biofilms have not been validated. We and others thus suggested exercising caution before the roles of biofilm formation in RVVC is excluded completely [19,20]. In further communications, Noverr and Fidel (2019), Swidsinski et al. (2019), and our group all agreed that visual proof of *Candida* biofilms on the human vaginal epithelium was needed to clarify this important question [[19], [20], [21]]. Using biopsy samples from patients with clinically and microbiologically diagnosed RVVC and VVC, and an array of methods that are highly sensitive and specific for *in vivo Candida* biofilms grown on vaginal tissues, this clinical study aimed to examine and characterise *Candida* growth in the vagina of RVVC patients. Biopsy samples were taken during an asymptomatic post-treatment period. Selecting such a period for the invasive biopsy procedure may reduce the risk of developing systemic infections, significantly increase patients’ willingness to participate in the study, and address the safety concern raised by the ethics committee. As RVVC and VVC often have same manifestations of disease during the acute infection period but differ significantly in their relapse patterns, selection of this asymptomatic period for biopsy was also to address our hypothesis that only RVVC patients harbor survivor *Candida* cells after antifungal treatment and these cells may repopulate and cause recurrent infections.

Ultimately, such a study deepens our understanding of *Candida* biological behaviour in the vagina that underpin the persistence of RVVC and will potentially improve the management of this troublesome infection.

## 2 Materials and methods

### 2.1 Ethics statement

**Fig. 1:**
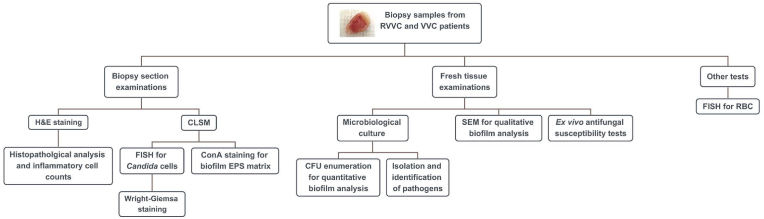
**Experimental design of the study.** RVVC, recurrent vulvovaginal candidiasis; VVC, vulvovaginal candidiasis; H & E, Hematoxylin–Eosin; CLSM, confocal laser scanning microscopy; FISH, fluorescence in situ hybridization; RBC, red blood cells; ConA, concanavalin A; EPS, extracellular polymeric substances; CFU, colony-forming unit; SEM, scanning electron microscopy.

This study followed the principles established in the Declaration of Helsinki and was approved by the Ethics Committee of Taizhou Hospital of Wenzhou Medical University (Approval numbers: K20210401 and K2022081). All experiments were carried out in accordance with the guidelines and regulations of the Committee. Written informed consent for participation was also obtained from patients. The overall study design was described in Fig. 1.

### 2.2 Recruitment of patients

More than 1000 women visited Taizhou Hospital of Wenzhou Medical University for VVC each year. One hundred and ten patients were approached for participation in this study between April 2020 and November 2022. Only six patients with clinically and microbiologically diagnosed RVVC and six with VVC agreed and were recruited. All participating subjects were outpatients and no hospital admission was required. Other underlying medical conditions of these patients were listed in Table 1. One RVVC patient was immunocompromised while all other participants had no known underlying diseases. The initial diagnosis of vulvovaginal candidiasis was based on evident signs/symptoms of vulvovaginitis and a positive *Candida* culture from patients’ vaginal discharge. All patients were followed up for at least 12 months after the initial hospital visit to determine whether the infection was VVC or RVVC. The number of infection episodes was determined by a participating gynaecologist. Caution was taken to differentiate a new episode from returning symptoms of an ongoing episode, based on the presence/absence of an entirely asymptomatic interval between the two consecutive “episodes”. Patients were given topical and/or oral antifungal therapies for symptomatic episodes; regimens for the most recent episode are listed in Table 1. For patients with polymicrobial infections (see Table 1), antimicrobials for non-*Candida* pathogens were also given. Microbiological tests were carried out to ensure that these patients were free of non-*Candida* vaginal infections before biopsy.

**Table 1:** Demographic and clinical information of participating patients.

Groups & patient ID	Age	Immune status	Number of episodes in the last 12 months	Monomicrobial or Polymicrobial ^+^	Fungal and other species isolated from vaginal swabs	Antifungal treatment for the latest symptomatic episode	Interval between the most recent treatment and biopsy *
**VVC**							
**PY1**	37	Competent	2	Monomicrobial	*C. albicans*	Clotrimazole vaginal tablet 500 mg, Q3D for 1 week	2 weeks
**PY2**	46	Competent	2	Monomicrobial	*C. albicans*	Clotrimazole vaginal suppository 160 mg, SID for 7 days	1 week
**PY3**	28	Competent	2	Polymicrobial	*C. albicans/M. genitalium*	Clotrimazole vaginal tablet 500 mg, Q3D for 1 week	3 days
**PY4**	44	Competent	2	Polymicrobial	*C. albicans/G. vaginalis*	Nifuratel (500 mg) and Nystatin (200,000 U) combinational vaginal capsule, SID for 5 days	3 days
**PY5**	30	Competent	2	Monomicrobial	*C. albicans*	Clotrimazole vaginal tablet 500 mg, Q3D for a week	1 week
**PY6**	46	Competent	1	Monomicrobial	*C. krusei*	Clotrimazole vaginal tablet 500 mg, Q3D for a week, then Q7D for 7 weeks	4 weeks
**RVVC**							
**QY1**	44	Competent	3	Polymicrobial	*C. albicans/M. genitalium*	Clotrimazole vaginal tablet 500 mg, Q3D for a week, then Q7D for 8 weeks	4 weeks
**QY2**	43	^#^Compromised	5	Monomicrobial	*C. albicans*	Clotrimazole vaginal tablet 500 mg, Q7D for 12 weeks	1 week
**QY3**	33	Competent	4	Monomicrobial	*C. glabrata*	Miconazole suppositories 200 mg, Q3D for 8 weeks	1 week
**QY4**	32	Competent	3	Polymicrobial	*C. albicans/G. vaginalis*	Clotrimazole vaginal tablet 500 mg, Q3D for a week	2 weeks
**QY5**	53	Competent	3	Monomicrobial	*C. albicans*	Clotrimazole vaginal tablet 500 mg, Q3D for a week	3 days
**QY6**	26	Competent	4	Monomicrobial	*C. glabrata*	Oral itraconazole 150 mg, SID for 2 days, followed by miconazole suppositories 200 mg Q3D for a week	16 weeks

+ Infections were categorized as monomicrobial if only a single *Candida* species was isolated from vaginal swabs, and polymicrobial if mixed cultures of *Candida* and pathogenic bacteria were isolated. # The patient was diagnosed with Sjögren's syndrome and received long-term prednisone at a dose of 10 mg SID. * We recommended an interval of 1–2 weeks between the most recent antifungal treatment and biopsy to all participating patients; the real time intervals varied due to patients' personal reasons. NA: Not applicable; *M. genitalium*: *Mycoplasma genitalium*; *G. vaginalis*: *Gardnerella vaginalis*; Q3D: once every three days; SID, once every day; Q7D, once every seven days.

### 2.3 Biopsy and tissue transportation

After confirming the diagnosis of RVVC or VVC, patients voluntarily underwent a vaginal biopsy during an asymptomatic period after antifungal therapies. Tissue samples of approximately 4 × 4 mm in diameter were taken from the vagina using clear plastic vaginal speculum and biopsy forceps (No. ER058R; Schubert, Tuttlingen, Germany). As all patients were at a post-treatment period, no evident white plaques were observed. Four samples were collected from different vaginal sites of the patient, including the posterior fornix (one from the top and one from 1 cm below), and the left and right lateral fornixes. Each sample was further sectioned into 2–3 smaller pieces for different experimental analyses (see Fig. 1). Biopsy samples were stored in sterile saline at room temperature and transferred to histopathological/microbiological laboratories for assessment within 24 h.

### 2.4 Qualitative and quantitative examinations of clinical biofilms

Fungal adherence to the vaginal mucosa was assessed qualitatively and quantitatively as previously described [22]. Biopsy tissues were washed with 0.9% saline to remove planktonic or loosely attached candida cells. High-resolution scanning electron microscopy (SEM) was used to visually examine the presence or absence of *Candida* growths on the vaginal tissue and their morphological characteristics, focussing on the vaginal epithelium. For SEM, vaginal tissues were fixed with 2.5 % (v/v) glutaraldehyde for 24 h at 4 °C and 1% osmium tetraoxide for 1 h at room temperature, and dehydrated with ethanol at gradually increased concentrations. Samples were coated with gold using a Balzers SCD005 sputter coater and viewed under a scanning electron microscope (Hitachi, S–300 N, Japan). Colony forming unit (CFU) enumeration was carried out to assess viable fungal burden in vaginal tissue. Vaginal tissues were homogenized using a tissue-homogenizer, followed by vortexing vigorously (30s × 4 times) and sonication at 42K Hz for 10 min. The suspensions were serially diluted with phosphate buffered saline (PBS), plated on Sabouraud's medium plates with chloramphenicol, and grown at 37 °C for 72 h for CFU counts per gram of vaginal tissue. To detect the presence of *Candida* biofilm extracellular polymeric substances (EPS), vaginal biopsy sections were stained with FITC-conjugated Concanavalin A (ConA, Invitrogen) at 1 mg/mL for 45 min [23] and examined with a confocal laser scanning microscope (CLSM, Nikon C2Si, Tokyo, Japan).

### 2.5 Fungal identification of *Candida* vaginal isolates

*Candida* clinical isolates from biopsies and grown on Sabouraud's medium plates for viable fungal burden assessment were identified to a species level using the following tests: chromogenic *Candida* medium (CHROMagar, Paris, France) and the Vitek matrix-assisted laser desorption/ionization-time of flight mass spectrometry (MALDI-TOF MS, bioMérieux, Craponne, France). These isolates were further used for *in vitro* biofilm formation and morphogenesis assessments.

### 2.6 Fluorescence in situ hybridization and fluorescent probes

Fluorescence in situ hybridization (FISH) was carried out on biopsy sections and *in vitro Candida biofilms* using the same method published by Swidsinski et al. (2019) [17]. 18S rRNA-based fluorescent probes Caal (GCC AAG GCT TAT ACT CGC T, for *C. albicans*) and PF2 (CTC TGG CTT CAC CCT ATT C, for other *Candida* spp.) and DAPI were all purchased from BioHub International (Shanghai, China). Mature *in vitro Candida* biofilms were grown on glass coverslips for 48 h, using RPMI-1640 (pH = 7.2) as the growth medium and RVVC clinical isolates [23]. Hybridized samples were viewed on Nikon C2Si confocal laser scanning microscope (Tokyo, Japan).

### 2.7 Wright-Giemsa staining and FISH for RBC

The same biopsy sections used in FISH with 18S rRNA probes were further stained with Wright-Giemsa stain for 15 min to detect the presence of human red blood cells (RBC) on these sections. A smear of RBCs and *C. albicans* cells stained with Wright-Giemsa stain served as controls. Hybridization of RBCs and 18S rRNA-based probes was also carried out to evaluate non-specific interactions between the probes and human RBCs.

### 2.8 Antifungal susceptibility testing

For planktonic cells, minimum inhibitory concentrations (MICs) of amphotericin B, fluconazole, 5-fluorocytosine, itraconazole, and voriconazole were determined using the ATB FUNGUS 3 kit (bioMérieux, Michaud, France), following the manufacturer's recommendations. This kit has a testing performance comparable to the broth microdilution method recommend by CLSI M27-A3 [24]. For epithelium-associated *Candida* biofilm cells, infected vaginal tissues were sectioned into small blocks of 2 mm × 2 mm x 2 mm and exposed to fluconazole at 8 μg/mL in RPMI-1640 for 24 h. The treated vaginal tissue blocks were washed and homogenized in PBS with a tissue homogenizer, and plated on Sabouraud's medium plates for viable fungal burden (37 °C, 48 h). Vaginal tissues treated with PBS only served as control.

### 2.9 Histopathological analysis

For histopathological study, infected vaginal tissues were fixed in 4% paraformaldehyde and tissue blocks were processed using a routine overnight cycle in a tissue processor. The tissue blocks were then embedded in wax and serially-sliced into 4 μm sections. The transverse sections were stained with Hematoxylin–Eosin (HE) for tissue damage, and viewed using a light microscope (LEICA DM 2000). The number of inflammatory cells in the microscope's field of view at 400× magnification was counted blindly by an experienced pathologist.

### 2.10 Data analysis and statistical methods

2-sample *t*-test was used to compare the means of inflammatory cell counts for different groups. Statistical significance was assumed at a *p* value of less than 0.05. Data analysis was performed using Minitab 16 software (Minitab, State College PA, USA).

## 3 Results

### 3.1 Demographic and clinical data of participating patients

Detailed demographic and clinical characteristics of recruited patients are shown in Table 1.

### 3.2 *Candida* species grew as morphologically disparate clinical biofilms on the vaginal epithelium of RVVC patients

**Fig. 2:**
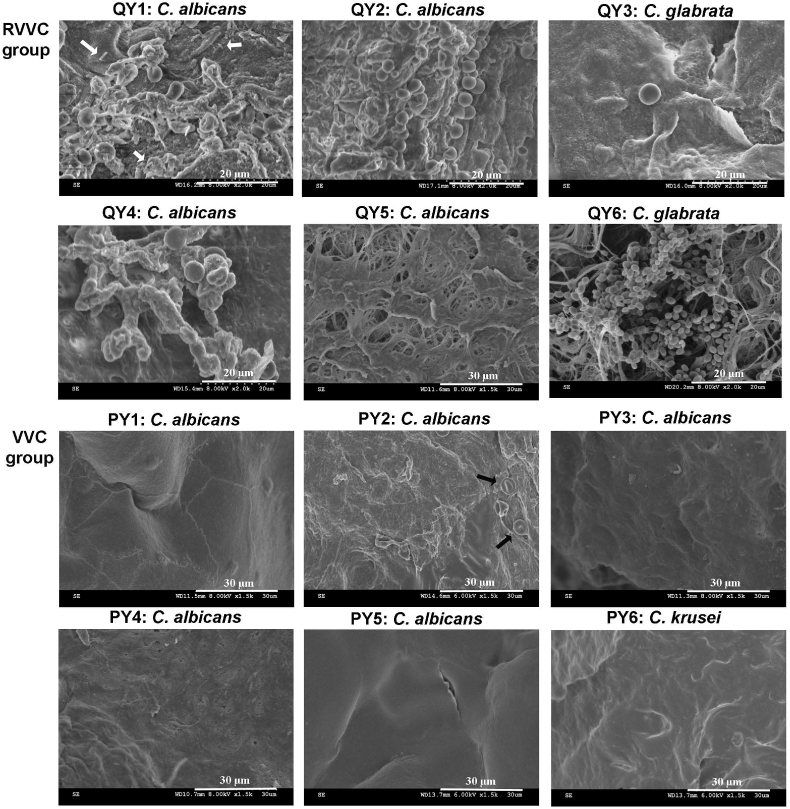
**Scanning electron microscopy showing adherent *Candida* growths on the vaginal epithelium of RVVC patients but not VVC patients** High-resolution scanning electron microscopy of vaginal tissues from patients with clinically confirmed RVVC showed different *Candida* adherent growths on the vaginal epithelium: Adherent monolayers (QY1 and QY2), microcolonies (QY4), adherent sporadic cells (QY3) and macro-colonies (QY6). Red blood cells (black arrows, PY2) and *Lactobacillus* cells (white arrows, QY1) were also observed, based on morphological analysis. No *Candida* cells were found on the healthy-looking vaginal epithelium of VVC patients or in RVVC patient QY5. Two biological samples from each patient were examined and representative images are shown for each patient. To be noted, biopsies were obtained from patients during a post-antifungal-treatment and asymptomatic period.

**Fig. 3:**
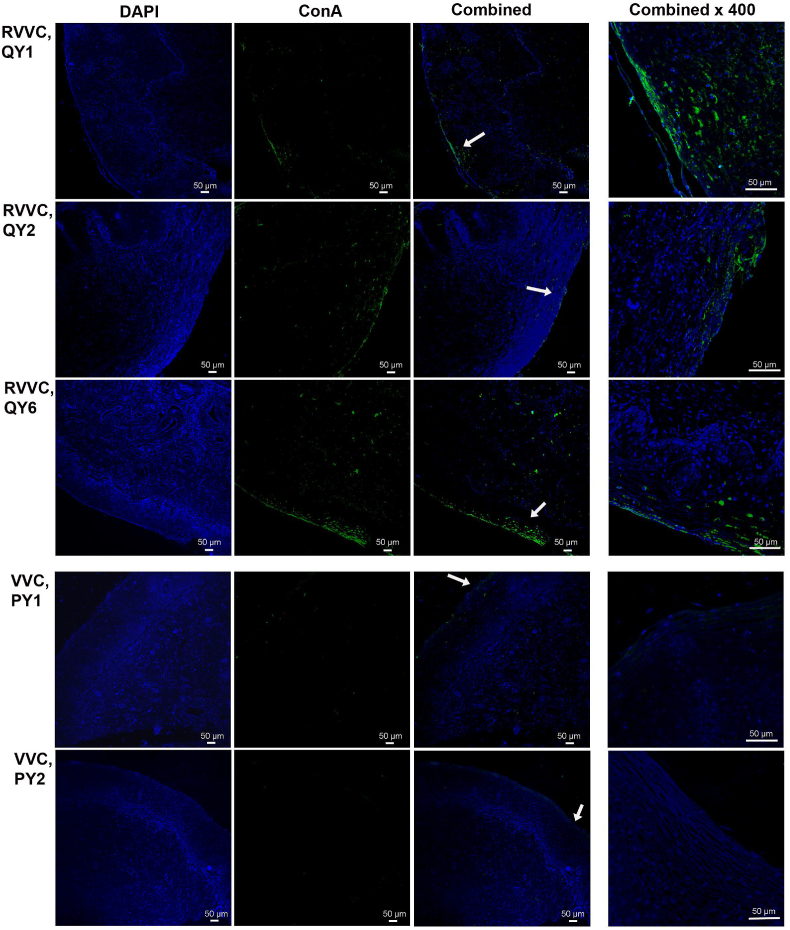
**Confocal laser scanning electron microscopy (CLSM) showing the presence of *Candida* biofilm extracellular polymeric substances (EPS) on the vaginal epithelium of RVVC patients** Vaginal biopsy sections from all RVVC and VVC patients were stained with DAPI (blue color, staining vaginal tissues) and FITC-labeled Concanavalin A (ConA, green color), and then examined by CLSM. ConA selectively binds to α-mannopyranosyl and α-glucopyranosyl glycoproteins and detects the presence of *Candida* biofilm EPS matrix. Strong ConA signals were found on the vaginal epithelium of RVVC patients, but not in VVC patients. Two biological sections from each patient were examined and only representative images from RVVC patients, QY1, QY2, and QY6 and VVC patients PY1 and PY2 were shown. The right column shows combined images of selected areas (white arrows) at a higher magnification (400×).

High-resolution SEM revealed *Candida* growing on the vaginal epithelium of five out of six RVVC patients, as sporadic yeast cells, adherent monolayers, and small (microcolonies) or large (macro-colonies) clusters of yeast cells (Fig. 2). Fungal growth on the vaginal epithelium appeared to be patchy, with most regions remaining free of *Candida* cells. CLSM using ConA staining detected *Candida* biofilm EPS on the vaginal epithelium in biopsy sections from RVVC patients, further supporting the presence of epithelium-associated *Candida* biofilms (Fig. 3). In contrast, no *Candida* cells or biofilm EPS were observed on the “healthy looking” vaginal epithelium of VVC patients after effective antifungal treatments (Fig. 2, Fig. 3).

Assessing viable fungal burden of washed and homogenized vaginal tissues quantitatively characterized epithelium-based clinical biofilms (Table 2). Fungal densities of infected tissues varied diversely and reached as high as ∼1 × 10^5^ CFU per gram of tissue. MALDI-TOF mass spectrometry results of infected vaginal tissues were in line with culture results of vaginal discharge from the microbiology diagnostic laboratory (Table 1, Table 2). No *Candida* cells were cultured from vaginal tissue from VVC patients or RVVC patient QY5 (Table 2).

**Table 2:** Quantitative analysis of *Candida* biofilms grown on the vaginal epithelium of RVVC/VVC patients^a^.

Patient groups	Patient IDs	Fungal cells isolated from vaginal tissues	Biofilm quantification (Fungal cell number/weight of tissue)
**RVVC**	QY1	*C. albicans*	5524 CFU/g
	QY2	*C. albicans*	5750 CFU/g
	QY3	*C. glabrata*	2197 CFU/g
	QY4	*C. albicans*	3252 CFU/g
	QY5	NA	0
	QY6	*C. glabrata*	104,645 CFU/g
**VVC**	PY1	NA	0
	PY2	NA	0
	PY3	NA	0
	PY4	NA	0
	PY5	NA	0
	PY6	NA	0

a Fungal densities are presented as the average of two tissue samples.

FISH combined with 18S rRNA-based probes was suboptimal in detecting *Candida* clinical biofilms grown on the vaginal epithelium.

**Fig. 4:**
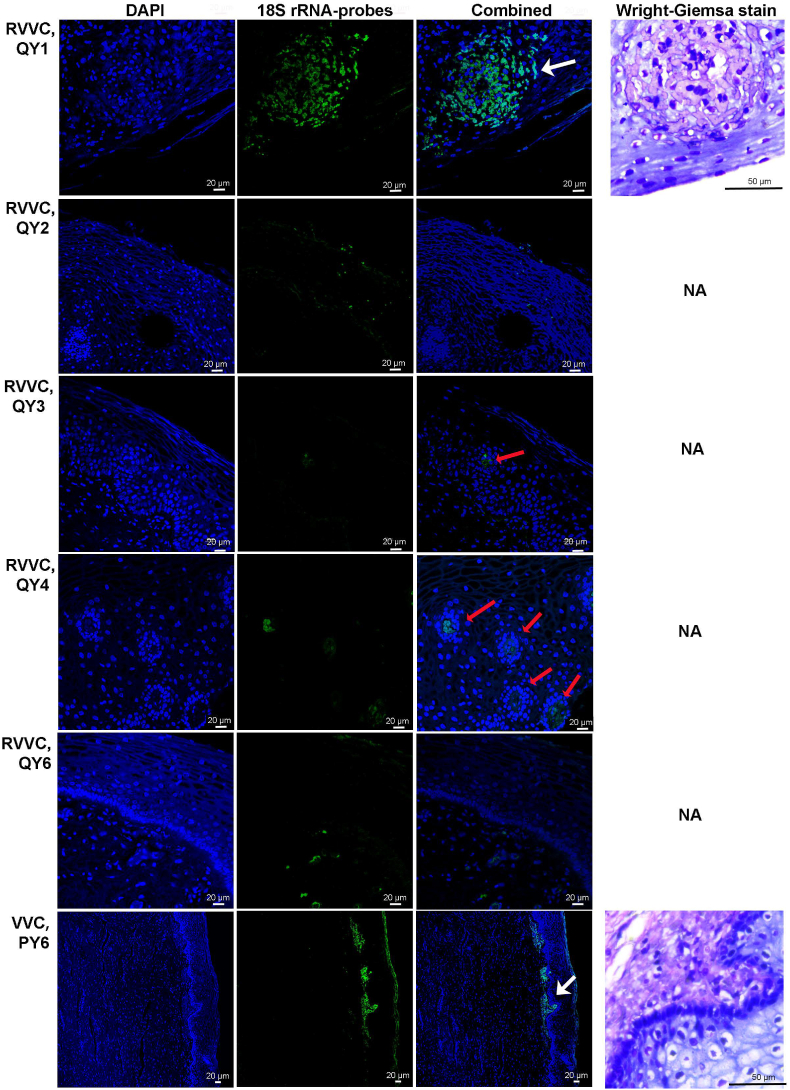
**Fluorescence in situ hybridization and Wright-Giemsa staining of vaginal biopsy sections from RVVC and VVC patients** DAPI was used to stain both human tissue and *Candida* cells and FITC-labeled 18S rRNA-based probes (Caal for QY1, QY2, and QY4, and PF2 for QY3, QY6 and PY6) were used to stain *Candida* cells in biopsy sections. Epithelial (QY2 and PY6) and sub-epithelial (QY1, QY3, QY4, QY6 and PY6) fluorescent signals of 18S rRNA-based probes were detected, suggesting the possible presence of *Candida* cells in vaginal tissue. The sub-epithelial 18S rRNA-based probe signals in QY3 and QY4 however, appeared to be overlapping with the blood vessels in the biopsy sections, indicating a possible cross-reaction between 18S rRNA-based probes and red blood cells (RBCs) residing in the subepithelial layer. Wright-Giemsa staining of the biopsy sections with strong subepithelial signals of 18S rRNA-based probes (QY1 and PY6) showed pink color of RBCs of the area of interest; *Candida* yeast cells stained by Wright-Giemsa stain often present purple color (see Fig. 5B). Shown are representative images of three independent repeats.

**Fig. 5:**
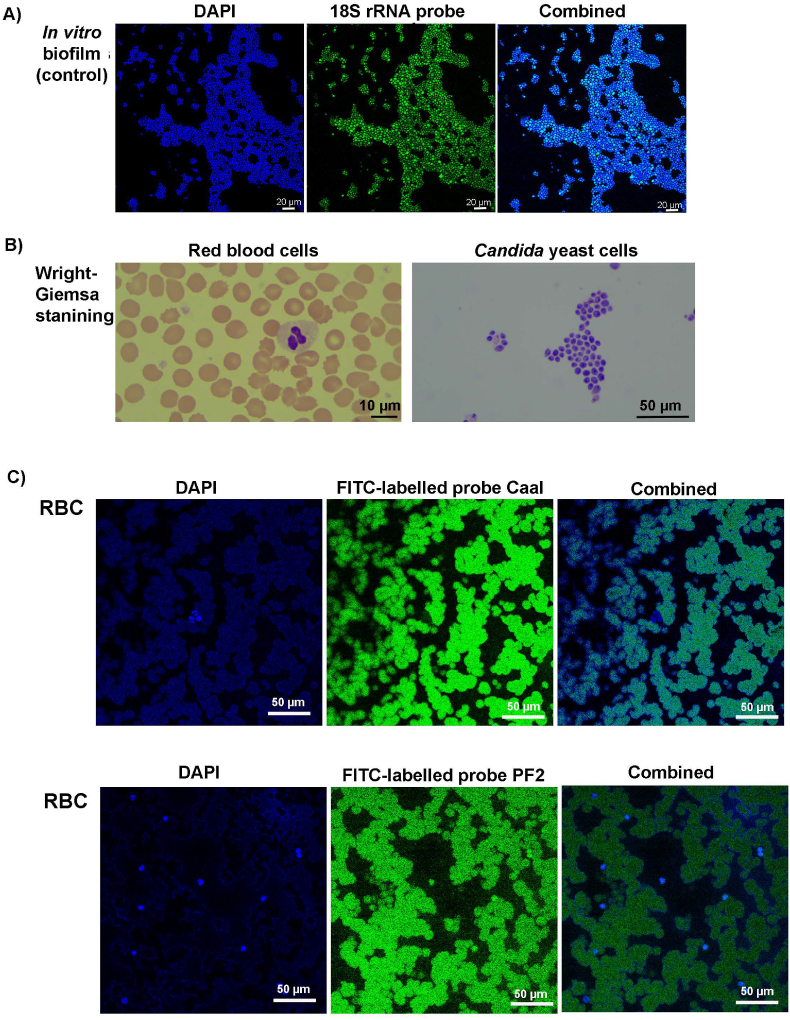
A) DAPI and 18S rRNA probe combinational staining of *in vitro Candida* biofilm formed by clinical isolates. *In vitro* biofilms of all clinical isolates were assessed and only that of QY1 were shown here; B) Wright-Giemsa staining of human red blood cells (RBCs, pink) and yeast cells of *C. albicans* clinical isolate QY1 (purple); these images serve as controls for Fig. 4; C) DAPI and FITC-labeled 18S rRNA-based probes (Caal and PF2) were used to stain smears of human RBCs. Strong fluorescent signals of probes were detected, indicating possible cross reactions between *Candida* 18S rRNA-based probes and RBCs. This experiment was carried out in three independent repeats and representative images were shown.

Fluorescent signals of 18S rRNA-based probes CaaI and/or PF2 were detected on the vaginal epithelium of RVVC patient QY2 and in the sub-epithelial space in most RVVC patients (Fig. 4). Surprisingly, fluorescent signals were also detected in the epithelium and sub-epithelial space of VVC patient PY6, despite no *Candida* cells was detected by SEM or recovered from microbiological culture. The subepithelial probe signals in RVVC patients QY3 and QY4 appeared to be in the blood vessels. While FISH combined with 18S rRNA-based probes was able to detect *in vitro* biofilms formed by *Candida* vaginal clinical isolates (Fig. 5A), it was also possible that 18S rRNA-based probes CaaI and PF2 bound non-specifically to human RBCs both within blood vessels and had escaped into the sub-epithelial space (Fig. 5). Investigatory Wright-Giemsa staining of the subepithelial areas with strong probe signals showed pink colours representing RBCs rather than *Candida* cells (white arrows in Fig. 4, Fig. 5B). FISH for RBC also confirmed non-specific binding of 18S rRNA-based probes (Fig. 5C). Further Homo sapiens BLAST also showed that the CaaI probe had partial complementarity to several human mRNA sequences including L3MBTL1, GFPT2, and ZNF737. The highest complementarity (14/19) was to L3MBTL1, which is expressed in the CD34^+^ hematopoietic stem/progenitor cells that give rise to RBCs. The PF2 probe also shares partial complementarity to human mRNA sequences including ALG14 and GFOD2. It is uncertain whether any of these mRNAs are present in mature RBCs. It does however, highlight that non-specific binding of the 18S rRNA-based probes is a possibility.

### 3.3 Histopathological analysis showed mild lymphocytic infiltration of the vaginal mucosa in RVVC

**Fig. 6:**
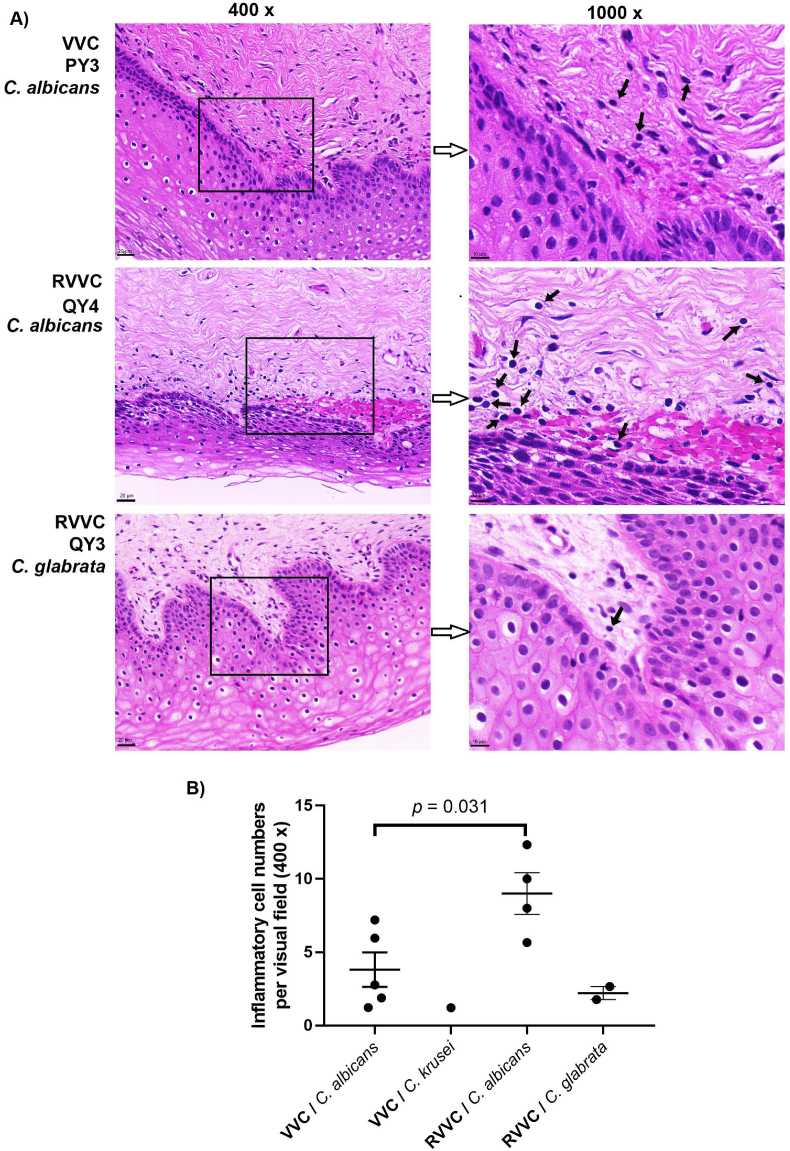
**Histopathological changes in the vagina of RVVC and VVC patients** Biopsies were obtained from patients during a post-antifungal treatment and asymptomatic period. Three biopsy sections from each VVC or RVVC patient were stained with Hematoxylin–Eosin (HE) and assessed for vaginal tissue damage. A) Mild histopathological changes were found for RVVC and VVC patients after antifungal treatments, presenting as lymphocytic infiltrations at the interface of the basal layer of the epithelium and lamina propria of the vaginal mucosa. No subepithelial microabscesses and neutrophil infiltration were observed for these patients. Images from representative patients PY3 (VVC caused by *C. albicans*), QY4 (RVVC caused by *C. albicans*) and QY3 (RVVC caused by *C. glabrata*) were shown. Right panels are selected areas of left panels with a higher magnification. Black arrows: lymphocytes. Scale bar: left panels, 20 μm; right panels, 10 μm. B) Patients with RVVC caused by *C. albicans* had significantly greater numbers of infiltrating lymphocytes in their vaginal mucosa than those with VVC by *C. albicans* (*p* = 0.031, using 2-sample *t*-test). Comparisons between patients with RVVC caused by *C. glabrata* or VVC by *C. krusei* and other groups were infeasible due to very small sample sizes. Three biopsy sections were randomly selected for each patient and three independent visual fields in each biopsy section were examined. The average cell count of nine visual fields of each patient was presented as a dot in the figure.

One of the key questions was whether the presence of epithelium-associated *Candida* cells was directly related to the pathological changes of the vagina. Haemotoxilin-eoxin staining showed mild lymphocytic infiltration at the interface of basal layer of the epithelium and the lamina propria in RVVC patients infected with *C. albicans* (Fig. 6A, black arrows). Patients who were infected with *C. glabrata* had even milder histopathological presentation, showing sporadic lymphocytes at the same interface. Cell counts further suggested significantly higher numbers of inflammatory cells in the vaginal mucosa of RVVC patients infected with *C. albicans*, relative to that of VVC patients after antifungal treatment (Fig. 6B).

### 3.4 Fluconazole at the highest serum concentration failed to eradicate *Candida* biofilms grown on the vaginal epithelium

Standard antifungal susceptibility tests designed for planktonic cells suggested that clinical isolates from RVVC patients remained mostly susceptible to first-line antifungal agents, such as amphotericin B, 5-FC, itraconazole, voriconazole and fluconazole (Table 3). CDC recommend oral fluconazole weekly for six months as the maintenance regimen for RVVC. *Ex vivo* susceptibility testing using infected vaginal tissues showed that fluconazole at the highest serum/tissue concentration achieved via oral administration, was able to inhibit further fungal growth but unable to effectively kill *Candida* cells grown on the vaginal epithelium as biofilms (Table 3).

**Table 3:** Antifungal susceptibilities of *Candida* spp. grown as planktonic cells and vaginal epithelium-based biofilms.

Strains	QY1 (*C. albicans*)	QY2 (*C. albicans*)	QY3 (*C. glabrata*)	QY4 (*C. albicans*)	QY6 (*C. glabrata*)
**Antifungals**	**Planktonic cells MIC (μg/mL)**^a^				
Amphotericin B	≤0.5	≤0.5	≤0.5	≤0.5	≤0.5
Interpretation^a^, ^b^	NA	NA	NA	NA	NA

Fluconazole	2	>128	≤1	≤1	≤1
Interpretation	S	R	SSD	S	SSD
5*-*fluorocytosine	≤4	≤4	≤4	≤4	≤4
Interpretation	NA	NA	NA	NA	NA
Itraconazole	0.25	>4	≤0.125	≤0.125	≤0.125
Interpretation	NA	NA	NA	NA	NA
Voriconazole	0.5	>8	≤0.06	0.125	≤0.06
Interpretation	I	R	NA	S	NA
**Fluconazole at 8 μg/mL**^c^	**Vaginal epithelium-based biofilm cells (CFU/g, percentage of survival)**				
Before treatment	4600 (100%)	5425 (100%)	3406 (100%)	3448,100%)	651,111 (100%)
After treatment	4255 (92.5%)	4067 (75.0%)	2322 (68.2%)	2414 (70.0%)	459,836 (89.2%)

a MICs were determined using ATB FUNGUS 3 kit (bioMérieux, Michaud, France).

b The Clinical & Laboratory Standards Institute (CLSI) clinical breakpoints were used to interpret MIC results for different antifungal agents and *Candida* species. Fluconazole for *C. albicans*: Susceptible ≤2; resistant >8. Fluconazole for *C. glabrata*: Susceptible-dose-dependent ≤32; resistant >64. Voriconazole for *C. albicans*: Susceptible ≤0.125; resistant >1. NA: CLSI clinical breakpoints not available.

c Fungal densities before and after treatment were determined and the average number of two samples was presented. 8 μg/mL is the highest concentration of fluconazole that can be achieved in serum or interstitial fluid via oral or systemic administration of this drug.

## 4 Discussion

Although formation of *in vivo* biofilms by *C. albicans* on the vaginal epithelium have been demonstrated extensively using murine VVC models [11,12], the presence of *Candida* biofilms in the human vagina and their role in the pathogenesis of VVC/RVVC was recently disputed [17,21]. Using tissue samples from patients who were otherwise healthy, we examined the growth modes of *Candida* in RVVC and its association with histopathologic changes of the vagina. Key findings of our study include: 1) *Candida* established morphologically disparate biofilms on the vaginal epithelium of RVVC patients; 2) epithelium-based biofilm growth of *Candida* was associated with mild lymphocytic infiltration of the vaginal mucosa; 3) biofilm growth led to persistence of epithelium-associated *Candida* under fluconazole treatment.

Clinical biofilms often lack the structural characteristics of *in vitro* biofilms [25,26]. Analysing vaginal tissues from RVVC/VVC patients with highly sensitive SEM and viable fungal burden assessment allowed us to clarify the pathogenic growth mode of *Candida* in the human vagina. For the first time, we found epithelium-associated *Candida* biofilms in the vagina of RVVC patients; the biofilm growth mode allowed *Candida* to survive fluconazole treatment and was associated with lymphocytic infiltration of the vaginal mucosa post-antifungal treatment. McKloud et al. (2021) examined the expression of key *Candida* biofilm-related genes in the vaginal lavage fluid and found higher expression of *HWP1*, *ECE1*, *ALS3* and *SAP* in patients with RVVC relative to that in healthy individuals [27]. These findings provided evidence to support the association between *Candida* biofilm growth and RVVC. The discrepancy in conclusions between our study and Swidsinski et al. (2019) [17] can be explained by different methodologies employed for sample analyses. We used an ample number of highly sensitive and specific methods including SEM, viable fungal burden, CLSM in combination with EPS-specific fluorescent staining, while Swidsinski et al. (2019) used a suboptimal FISH with 18S rRNA-based probes to examine clinical *Candida* biofilms grown on human vaginal epithelium [17].

Although clinical and *in vitro* biofilms often have distinct and unique morphologies, they share an important trait of high-population-density that underpins their antimicrobial resistance [25,28]. We examined the vaginal tissues of RVVC patients and found patchy growth of *Candida* biofilms on the vaginal epithelium. Such a confined growth of *Candida* cells on the vaginal epithelium may result in a local population with a density significantly higher than its log-planktonic counterpart. Resistance and tolerance of *Candida* biofilms to antifungal drugs are known to be multifactorial. In addition to the extracellular matrix that provides a physical barrier for antifungal drugs or causes drug sequestration, upregulation of drug-efflux pumps, and the presence of tolerant and persister cells, antifungal resistance of *Candida* biofilms can also be attributed to increased cell density of this special growth mode, upregulated general stress responses within biofilms, and quorum sensing systems [28,29]. Unlike that of RVVC patients, no *Candida* biofilms were detected in the vagina of VVC patients after antifungal treatments. It is possible that *Candida* cells in VVC patients remain a “free-living” state and susceptibility to antifungal treatment, or VVC patients themselves had genetic susceptibilities that facilitate the eradication of fungal cells.

Tolerance of epithelium-based *Candida* biofilms to fluconazole at the highest serum concentration achieved by oral administration of fluconazole tablet may explain why CDC-recommended long-term maintenance therapies are able to promote an extended asymptomatic condition for RVVC patients but are rarely curative, despite the *in vitro* sensitivity of *Candida* isolates to fluconazole [2]. Oral administration of fluconazole was found to result in similar fluconazole levels in plasma and vaginal secretions; a 150 mg single oral dose can lead to 2.82 μg/mL fluconazole in the serum and 2.43 μg/mL in vaginal secretions [30]. The presence of *Candida* biofilms in the human vagina implicates that antifungal therapies targeting biofilms rather than planktonic cells may be considered for RVVC patients in the future [26].

Micro-abscesses and neutrophil infiltration, two histopathological changes often found in the murine model of VVC, were not observed in RVVC patients after antifungal treatment [11,12]. While the current rodent model of VVC may represent the acute stage of human VVC/RVVC, it does not reflect the inflammatory status of the chronic phase of RVVC. Therefore, a new animal model is needed for comprehensive understanding of the disease pathogenesis and to develop more effective therapies. Using less virulent vaginal *Candida* isolates, prolonging the use of exogenous estrogen, and extending the infection period from 48-72 h to 1–2 weeks may re-direct the diseased from neutrophil-dominated acute infections to lymphocyte-dominated chronic infections.

We acknowledge that this study still suffers from several limitations. One evident limitation of our study was the relatively small number of patients recruited owing to unwillingness of patients to undergo an invasive procedure and high labour-intensity required to analyse each tissue sample. Due to the small sample size, the conclusion drawn from this study may not fully represent infections caused by other *Candida* isolates. Biopsy is not a routine clinical practice for patients with RVVC due to the risk of developing systemic infections and patients’ reluctance. In this study, biopsies were taken at a post-antifungal-treatment period, as previously described. It is possible that biofilms formed by *Candida* spp. on the vaginal epithelium during a fully active and symptomatic infection have different morphological characteristics, most likely showing more “typical” biofilm structures. Another limitation is lack of biopsy samples from healthy participants to demonstrate the difference between a disease condition and healthy colonization, again due to low willingness of participants to undertake an invasive biopsy procedure. The strengths of this study were the strict selection of RVVC and VVC patients, and using highly-sensitive qualitative and quantitative methods to examine infected vaginal tissues. All patients were seen and followed up by a participating gynaecologist for at least 12 months to ensure an accurate diagnosis. Inspired by Swidsinski et al. (2019) [17], we used vaginal biopsy samples, high-resolution SEM, CLSM in combination with EPS staining, and viable fungal burden assessment to study the interaction between *Candida* and the human vaginal epithelium; such a combination has adequate sensitivity for a minimum presence of *Candida* biofilms in the human vagina.

## 5 Conclusions

In summary, our study provided direct evidence to support the involvement of *Candida* biofilms in the pathogenesis and persistence of RVVC.

## Ethics approval and consent to participate

This study was approved by the Ethics Committee of Taizhou Hospital of Wenzhou Medical University (Approval numbers: K20210401 and K2022081). Written informed consent for participation was also obtained from patients.

## CRediT authorship contribution statement

**Yihong Pan:** Funding acquisition, Methodology, Validation. **Yao Sun:** Methodology, Validation. **Lanqian Chen:** Methodology, Validation. **Yali Cheng:** Methodology, Validation. **Panpan Jin:** Methodology, Validation. **Weidan Zhang:** Methodology, Validation. **Lingzhi Zheng:** Methodology, Validation. **Junyan Liu:** Methodology, Validation. **Tieli Zhou:** Methodology, Validation. **Zhenbo Xu:** Conceptualization, Funding acquisition. **Cheng Li:** Methodology, Validation. **Xenia Kostoulias:** Methodology, Validation, Writing – review & editing. **Cathy J. Watson:** Methodology, Validation, Writing – review & editing. **David McGiffin:** Conceptualization, Writing – review & editing. **Anton Y. Peleg:** Conceptualization. **Yue Qu:** Conceptualization, Funding acquisition, Writing – original draft, Writing – review & editing.

## Declaration of competing interest

The authors declare the following financial interests/personal relationships which may be considered as potential competing interests:Yue Qu reports financial support was provided by The Alfred Research Trusts. Yihong Pan reports financial support was provided by Zhejiang Province Natural Science Foundation. Zhenbo Xu reports financial support was provided by South China University of Technology.

## Data Availability

Data will be made available on request.
